# Application of Microfluidic Chips in Separation and Analysis of Extracellular Vesicles in Liquid Biopsy for Cancer

**DOI:** 10.3390/mi10060390

**Published:** 2019-06-11

**Authors:** Jin Lu, Jiushen Pang, Ying Chen, Qi Dong, Jiahao Sheng, Yong Luo, Yao Lu, Bingcheng Lin, Tingjiao Liu

**Affiliations:** 1 College of Stomatology, Dalian Medical University, Dalian 116044, China; phoenixluj@163.com (J.L.); pjs3333@163.com (J.P.); cy527744719@163.com (Y.C.); Dongqi9277@163.com (Q.D.); sjh827756903@163.com (J.S.); 2 Faculty of Chemical, Environmental and Biological Science and Technology, Dalian Technology University, Dalian 116044, China; yluo@dlut.edu.cn; 3 Department of Biotechnology, Dalian Institute of Chemical Physics, Chinese Academy of Sciences, Dalian 116044, China; luyao@dicp.ac.cn (Y.L.); bclin@dicp.ac.cn (B.L.)

**Keywords:** Microfluidic, extracellular vesicles, cancer, liquid biopsy

## Abstract

Extracellular vesicles (EVs) are becoming a promising biomarker in liquid biopsy of cancer. Separation EV from cell culture medium or biofluids with high purity and quality remains a technique challenge. EV manipulation techniques based on microfluidics have been developed in the last decade. Microfluidic-based EV separation techniques developed so far can be classified into two categories: surface biomarker-dependent and size-dependent approaches. Microfluidic techniques allow the integration of EV separation and analysis on a single chip. Integrated EV separation and on-chip analysis have shown great potential in cancer diagnosis and monitoring treatment of responses. In this review, we discuss the development of microfluidic chips for EV separation and analysis. We also detail the clinical application of these microfluidic chips in the liquid biopsy of various cancers.

## 1. Introduction

Currently, tissue biopsy is still the standard procedure of cancer diagnosis. However, several limitations of tissue biopsy exist [1,2]. Firstly, tissue biopsy may not accurately reflect the global features of the examined case because of the spatial heterogeneity within a single tumor mass. Secondly, tissue biopsy cannot monitor the dynamic changes of a tumor in real time because it cannot be performed multiple times. Especially, there is not enough tumor tissue for biopsy in some patients, such as postoperative cases. Thirdly, tissue biopsy is invasive and brings some risks to patients. In addition, it is costly and time-consuming. Thus, it is necessary to develop new approaches to overcome these limitations of tissue biopsy.

Liquid biopsy is a new technique that detects cancer cells or their products in the body fluids of cancer patients [3]. Compared with tissue biopsy, liquid biopsy may better reflect the tumor complexity and heterogeneity as a whole. Liquid biopsy can be performed multiple times, thus revealing tumor evolution nearly in real time [4]. It is becoming a revolutionary technique that provides genomic and proteomic information about patients with cancer [5]. The components assessed by liquid biopsy commonly include circulating tumor cells (CTCs) [6,7,8], circulating tumor DNA (ctDNA) [9,10], and extracellular vesicles (EVs) [11,12,13]. Compared with CTCs and ctDNA, EVs are more likely to reflect a role in cell-to-cell communications [14]. EVs are becoming potential cancer biomarkers for early detection, diagnosis, prognosis, and even therapy selection [15]. 

## 2. Extracellular Vesicles (EV)

**Figure 1:**
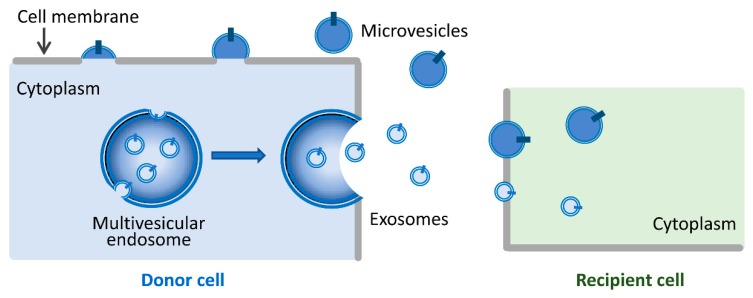
A schematic diagram depicting exosome and microvesicle generation and EV-mediated cell–cell communications. Exosomes are secreted upon fusion of multivesicular endosomes with the cell surface. Microvesicles are generated by the outward budding and fission of the cell membrane. Both exosomes and microvesicles can transfer their cargos from donor cells to recipient cells and consequently modulate recipient cell function.

EVs are nanosized, membrane-bound vesicles released from cells that can transport cargos between cells [16]. Based on their biogenesis, content, and secretory pathways, EVs can be divided into two broad categories: exosomes (30–100 nm) and microvesicles (100–1000 nm) [13,14,16,17,18]. As shown in Figure 1, exosomes are secreted upon fusion of multivesicular endosomes with the cell surface [19,20], while microvesicles are generated by the outward budding and fission of the plasma membrane and the subsequent release of vesicles into the extracellular space [21]. Until now, there have been no specific markers to differentiate EV subtypes. It remains extraordinarily difficult to assign an EV to a particular biogenesis pathway unless the EV is caught in the act of release by live imaging techniques. In this review, we use the term EVs to represent exosomes and microvesicles.

EVs were initially described as a means of eliminating unneeded compounds from the cell. Although Poste et al. suggested that EVs played a regulatory role in tumor cells in 1980 [22], little attention was paid to these vesicles for a long time. In 1983, Pan et al. and Harding et al. consecutively showed that proteins were selectively packaged into vesicles and that EVs might play important roles in biological processes [23,24]. A very important study in the EV research field by Valadi et al. [25,26,27,28] showed that both mRNA and microRNA were found in EVs and could be delivered to other cells. After that, many more studies demonstrated the physiological and pathological functions of EVs [29]. We now know that EVs carry a diverse variety of functional proteins, lipids, microRNAs, mRNAs, long non-coding RNAs, and occasionally genomic DNA. These biomolecules are important mediators for short or distant cell to cell communications [30]. EVs are increasingly being recognized as important vehicles in cellular communications, triggering phenotypic changes in receptor cells [14]. 

EVs are abundant in blood and also present in other body fluids, such as urine, saliva, and cerebrospinal fluid [31,32]. Collection of these body fluids is minimally invasive or non-invasive. Studies of EV-based liquid biopsy are increasing. It is known that tumor cells secret more EVs than normal cells [33]. Higher concentration of EVs was detected in serum of cancer patients than of healthy individuals [34]. EVs could help to distinguish early and late stage cancer patients [34,35]. Furthermore, EVs contain numerous bioactive molecules (mRNA, miRNA, DNA, lncRNA, and proteins). Thus, EVs are potential biomarkers for cancer diagnosis and prognosis [31]. For example, Skog et al. suggested that microvesicles derived from blood could provide information for glioblastoma diagnosis [36]. Hannafon et al. reported that exosomal miR-1246 and miR-21 are significantly upregulated in plasma of breast cancer patients [37]. Matsumura et al. identified miR-19a in serum exosomes as a prognostic biomarker for recurrence in colorectal cancer patients [38]. 

Separation of EV from cell culture medium or biofluids with high purity and quality remains a challenge. The terms “separation” and “concentration” are commonly used [39]. Separation refers to purification or isolation of EVs from other non-EV components of the materials (conditioned medium, biofluid, tissue) and the different types of EVs from each other. Concentration is a means to increase numbers of EVs per unit volume, with or without separation. Currently, EV separation and concentration can be achieved by multiple technologies based on EV size or surface marker expression. These techniques include differential ultracentrifugation, density gradient centrifugation, immunoaffinity, ultrafiltration, polymer-based precipitation, and size-exclusion chromatography [17]. Differential centrifugation is the most common approach for EV separation. Usually, samples are first centrifuged at low speed to remove cells (500× *g*). Then, cell debris is removed after centrifugation at 2500× *g*. The supernatant is collected and then centrifugation is performed at 10,000× *g* to pellet large EVs, such as microvesicles. The final supernatant is then ultracentrifuged at 100,000× *g* to pellet the small EVs that correspond to exosomes. The final pellet is then washed in a large volume of phosphate buffered solution (PBS) to eliminate contaminating proteins, then centrifuged one last time at 100,000× *g* [40,41,42]. To achieve better specificity of EV or EV subtype separation, most researchers use one or more additional techniques. Density gradient centrifugation (velocity or flotation) could further improve EV purity. Exosomes are purified in a buoyant density using a discontinuous gradient of a sucrose solution or iodixanol cushion [43,44]. Additional purification can be achieved by immunoaffinity as well. Antibodies (CD63, CD81, CD9) are usually conjugated with magnetic beads and incubated with EV-containing samples [45]. EVs can be separated by ultrafiltration based on their size. The most common filter pore sizes are 0.8 μm and 0.22 μm [40]. Some commercial products also use polyethylene glycol (PEG) for precipitation to isolate EVs [46]. Size-exclusion chromatography separates EV particles by their sizes [47]. To confirm the purity of separated EVs electron microscopy, nanoparticle tracing analysis (NTA), and western blotting are usually performed to characterize EV shape, size, and biomarker expression [48]. Usually, at least three positive protein markers (such as CD63, CD9, CD81, TSG101, etc.) and a negative protein marker are necessary (such as calnexin) to define EVs. A single EV could be characterized through two different but complementary techniques: microscopy (such as scanning-probe microscopy, atomic force microscopy, or super-resolution microscopy) or single particle analyzers (NTA, high resolution flow cytometry, and dynamic light scattering) [49].

## 3. Microfluidic Chips for EV Separation and Analysis

Conventional EV separation methods are time consuming and require a large sample volume. Microfluidic technologies have aroused a great interest in the biological and medical sciences due to their ability to precisely manipulate small volume liquid samples, reduced operation time, and for their low cost [50]. They have been widely used in biomedical research, point-of-care diagnosis, analytical chemistry, and environmental monitoring [51,52,53,54,55]. They provide fast, efficient, compact solutions for fluid-related problems. In recent years, microfluidic techniques have been successfully applied for the separation of micro- and nanoparticles [56,57]. Over the last two decades, microfluidic technologies for separating CTCs have been developed based on various physical properties or surface biomarkers of CTCs [4,58], while EV manipulation techniques based on microfluidics have been developed in the last decade. As EVs are smaller than CTCs, nanofabrication techniques have presented more important roles in size-dependent EV separation. A variety of microfluidic devices have been presented for both EV separation and detection (Table 1). These devices show great potential in cancer diagnosis and monitoring of treatment responses. Microfluidic-based EV separation techniques developed so far can be classified into two categories: surface biomarker-dependent and size-dependent approaches.

### 3.1. Surface Biomarker-Dependent Separation

#### 3.1.1. Immunoaffinity Separation via Solid Surfaces

**Figure 2:**
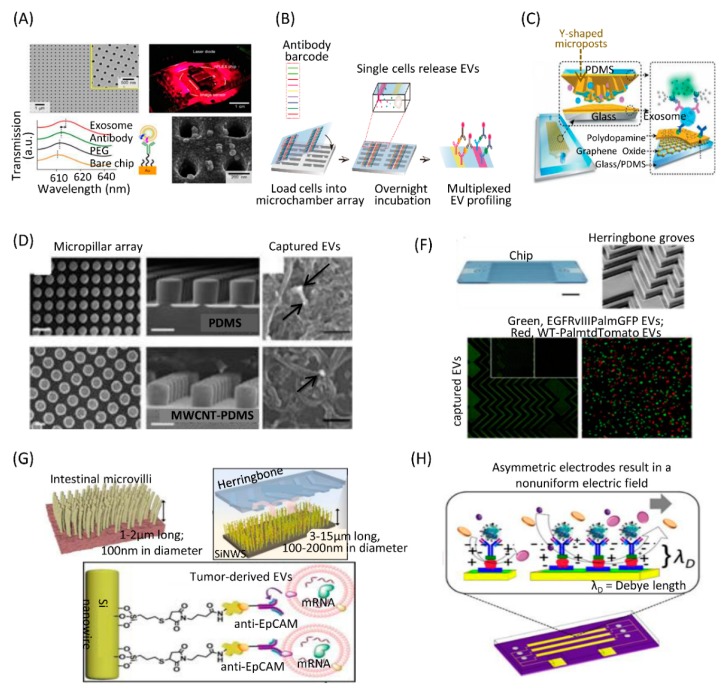
Immunoaffinity separation via solid surfaces on microfluidic chips. (**A**) Label-free detection of immunocaptued EVs with the nPLEX sensor. Reprinted with permission [60]. Copyright 2014, Nature Publishing Group. (**B**) Platform for multiplexed profiling of single-cell EV. Reprinted with permission [61]. Copyright 2019, NAS Publishing Group. (**C**) A microfluidic platform containing Y-shaped microposts coated with graphene oxide (GO) and polydopamine (PDA) as a nanostructured interface for the sandwich ELISA of EVs with enzymatic fluorescence signal amplification. Reprinted with permission [62]. Copyright 2016, Royal Society of Chemistry Publishing Group. (**D**) A microfluidic chip containing PDMS micropillar arrays functionalized with MWCNTs. Reprinted with permission [63]. Copyright 2017, American Chemical Society Publications. (**E**) A microfluidic chip with herringbone structures coated with a cocktail of antibodies. Reprinted with permission [65]. Copyright 2018, Nature Publishing Group. (**F**) A microfluidic chip with herringbone and bionic microvilli structures coated with anti-EpCAM antibodies. Reprinted with permission [67]. Copyright 2019, ACS Publications. (**G**) A microfluidic device with tunable alternating current electrohydrodynamics to induce fluid flow vortices and micromixing in microchannels. Reprinted with permission [69]. Copyright 2014, ACS Publications.

The most commonly used microfluidic approach for EV separation is the capture of specific EVs using antibodies immobilized in microfluidic chips. As most EVs express CD63, CD9, and CD81 on their membrane surface, these antibodies are widely used to capture EVs. Kanwar et al. developed a high throughput microfluidic chip that allowed simultaneous capture and quantification of EVs in a single device. The chip contained 12 channels placed laterally. Each channel was 73 mm in length and 100 μm in height, with eight circular chambers. The chip was pre-coated with EV-specific capture antibodies (anti-CD63). Subsequent EV detection could be achieved with a fluorescent carbocyanine dye (DiO) [59]. Im et al. presented a microfluidic chip based on surface plasmon resonance (SPR)-based assay for label-free, high-throughput EV analyses (Figure 2A). This nano-plasmonic exosome (nPLEX) sensor contained periodic nanoholes. Antibodies anti-CD63 and anti-CD24 were conjugated to the sensor surface to capture EVs [60]. Recently, our group developed a high throughput single-cell EV capture and detection microfluidic platform based on immunoaffinity approach (Figure 2B). This system consisted of two components: a high-density microchamber array and an antibody barcode glass slide. The microchamber array contained 6343 identical units for single cell culture, and the antibody barcode array consisted of 9 parallel microchannels coated with different antibodies for EV capture and detection. Based on this high-throughput multiplexed platform, we revealed the single-cell heterogeneity of EV secretion ability [61]. 

To enhance the capture efficiency for the EVs on microfluidic chips, researchers designed nanostructures on chips to provide a larger surface area that allows direct incorporation of capture antibodies. Zhang et al. developed a microfluidic platform containing an array of Y-shaped micropillars to increase contact area and create turbulence flow (Figure 2C). Antibodies were immobilized on the surface of these nanostructures. This platform was able to efficiently capture EVs and discriminate specific EVs derived from ovarian cancer patients from the healthy controls by the quantitative detection of three biomarkers (CD63, CD81, and Epithelial Cell Adhesion Molecule (EpCAM)) [62]. Wang et al. developed a microfluidic chip containing Polydimethylsiloxane (PDMS) micropillar arrays that were chemically functionalized with Multiwall carbon nanotubes (MWCNTs) by an electroless plating method (Figure 2D). Then, biotinylated anti-CD63 antibody was conjugated with the MWCNT device for immuno-capture of EVs. The micropillar array and unique topography of the nanomaterials significantly increased the contact area and enhanced interactions between EVs and capture antibodies [63].

Another strategy to enhance EV capture efficiency is by enhancement of the fluidic whirling as the fluid travels through microchannels. Herringbone nanostructures are often used to create anisotropic flow in microchannels to produce micro-vortices that promote interactions between the EVs and the antibody-coated surface of the microfluidic device, thereby enhancing binding of EVs to the microchannel surface. Chen et al. first presented an approach to immunocapture EVs from cell culture medium and blood samples on a microfluidic chip. The device was a straight flow channel of 19 mm width, 20 mm depth, and 4.5 cm length with herringbone groves on its ceiling. Anti-CD63 antibody was coated on the microfluidic channel surface. EVs from glioblastoma cell culture supernatant were captured in the microchannels [64]. Reátegui et al. developed a high-throughput herringbone microfluidic chip, in which the surface was coated with a cocktail of antibodies (Figure 2E). The chip captured EVs derived from glioblastoma multiforme (GBM) patient serum or plasma [65]. Hisey et al. developed a herringbone-grooved microfluidic device that was coated with anti-CD9 or anti-EpCAM antibody to capture EVs from high-grade serous ovarian cancer serum [66]. Dong et al. developed a herringbone microfluidic chip with nanowire arrays to mimic the distinctive structures of intestinal microvilli (Figure 2F). Combining herringbone with microvilli dramatically increased the surface area and enhanced EV capture efficiency [67]. Kang YT et al. developed a dual-patterned immunofiltration microfluidic chip for specific EVs capture. This device consisted of two distinct immuno-patterned layers that were able to generate the vortex flow to improve the chance of interactions between the antibody and EVs [68]. Vaidyanathan et al. developed a microfluidic device that used tunable alternating current electrohydrodynamics to induce fluid flow vortices and the associated micromixing in microchannels (Figure 2G). This approach enhanced the specificity of capture and also reduced nonspecific adsorption. The device showed a 5-fold increase in capture and detection performance in comparison to hydrodynamic fluid [69].

#### 3.1.2. Immunoaffinity Separation via Magnetic Nanoparticles

**Figure 3:**
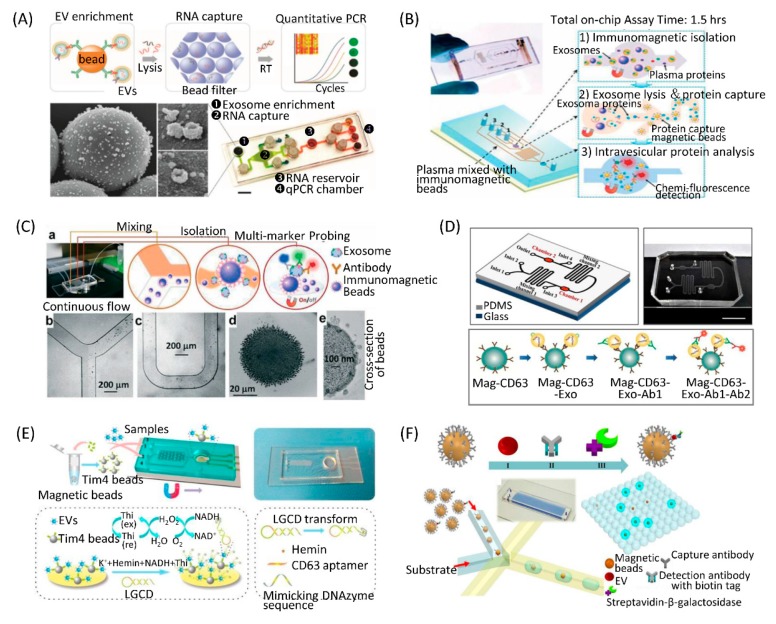
Immunoaffinity separation via magnetic nanoparticles on microfluidic chips. (**A**) A microfluidic platform containing EV enrichment and EV RNA analysis units. Reprinted with permission [70]. Copyright 2015, Nature Publishing Group. (**B**) A microfluidic device integrating EV capture with immunomagnetic beads and analysis by on-chip ELISA. Reprinted with permission [71]. Copyright 2014, RSC Publishing Group. (**C**) A microfluidic chip combining EV capture with immunomagnetic beads and analysis by fluorescent imaging. Reprinted with permission [72]. Copyright 2016, RSC Publishing Group. (**D**) A microfluidic device capturing EVs with anti-CD63 immunomagnetic beads and quantifying EVs with immunofluorescent staining. Reprinted with permission [73]. Copyright 2017, PLOS Publications. (**E**) A microfluidic chip featuring Y-shaped microcolumns to enhance EV labeling by Tim4 beads and ITO electrodes to detect EVs by LGCD transformation. Reprinted with permission [74]. Copyright 2018, ACS Publications. (**F**) A droplet digital ExoELISA platform for single-EV-counting immunoassays. Reprinted with permission [76]. Copyright 2018, ACS Publications.

Compared to the microfluidic chips with immobilized antibodies on the channel surfaces, the immunomagnetic bead allows for higher EV capture efficiency due to the larger surface area and convenient sample preparation in the following analysis. Shao et al. developed a microfluidic platform that integrated three functional compartments: An EV enrichment chamber, RNA extraction chamber, and RNA analysis chamber (Figure 3A). They used immunomagnetic beads coated by anti- epidermal growth factor receptor (EGFR) or anti-EGFRvIII antibody to capture EVs obtained from the blood of GBM patients. Captured EVs were lysed and mRNA contents were analyzed in the RNA analysis chamber on the chip [70]. He et al. designed a microfluidic chip to separate EVs from patient plasma using immunomagnetic beads (Figure 3B). They integrated EV capture and analysis on a single chip. The captured EVs were lysed and the released intravesicular proteins were detected by detection antibodies and chemifluorescence reagents. This approach captured subpopulation EVs and quantitatively analyzed both surface and intravesicular proteins from a small volume of samples within 100 min [71]. Zhao et al. designed a simple microfluidic chip to isolate EVs for in situ and multiple detection using immunomagnetic beads (Figure 3C). This chip consisted of a Y-shaped channel, a winding mixer, and a reservoir. They applied the chip for detection of ovarian cancer plasma EVs by multiplexed measurement of three EV biomarkers (CA-125, EpCAM, CD24) correlated with tumor existence [72]. Our group developed a microfluidic chip in which EVs could be captured by immunomagnetic beads from breast cancer cell conditioned medium or patient plasma (Figure 3D). Using this device, we found that the number of EpCAM-positive EVs was significantly increased in the plasma of breast cancer patients than healthy controls [73]. Xu et al. developed a two-stage microfluidic chip that integrated on-chip isolation and in situ electrochemical analysis of EVs (Figure 3E). As the Tim4 protein can specifically conjugate with phosphatidylserine expressed on EV membranes, magnetic beads were coated with Tim4 to label EVs. At the EV isolation stage of the chip, PDMS Y-shaped micropillars were designed to enhance the contact opportunities between Tim4-modified magnetic beads and EVs. Then, the captured EVs were detected sensitively through magnetic enrichment on the surface of the ITO electrode, where a new signal transduction strategy was constructed [74]. 

Differing from capture of EVs using immunomagnetic beads, Ko et al. developed an EV track-etched magnetic nanopore chip to separate EVs. Before introduction into the chip, EVs were magnetically labeled with streptavidin-coated 50 nm magnetic nanoparticles and biotinylated antibodies (CD63, CD9, CD81, or EpCAM). Magnetically labeled EVs experienced two forces when they passed through the nanopores—a drag force from the fluid flow and a magnetophoretic force from the pore’s edge, where the magnetic field gradient was maximized. When the magnetic trapping force overcame the drag force from the fluid flow, EVs were trapped at the edge of the magnetic nanopores [75].

The study of single EVs is still technically challenging. Liu et al. developed a single-exosome-counting enzyme-linked immunoassay (droplet digital ExoELISA) for EV quantification (Figure 3F). A single EV was immunocaptured on a magnetic bead. Substrates and beads were co-encapsulated into microdroplets on a microfluidic chip. For those droplets that contained the beads with the EV immunocomplex, the substrate was catalyzed by the enzyme to emit fluorescein within the droplets. The droplet-based single-EV-counting enzyme-linked immunoassay was able to achieve a limit of detection down to 10 enzyme-labeled EV complexes per microliter [76]. 

### 3.2. Size-Dependent Separation

#### 3.2.1. Filtration Using Nanoporous Membrane

Ultrafiltration is a size dependent EV separation technique. In most protocols, samples are driven by pressure through a filter with 200 nm–800 nm pore-size to separate small and large EV subpopulations. Liu et al. developed an exosome total isolation chip (ExoTIC) that used a simple filtration approach, in which EV-containing samples were passed through a nanoporous membrane to enrich and purify intact EVs in the 30–200 nm size range. Small molecules, such as free nucleic acids, proteins, and lipids, were flushed out. They found that certain miRNAs were more highly expressed in EVs isolated by ExoTIC compared to ultracentrifugation, and vice versa. Clinical samples tested on this chip included plasma, urine, and lung bronchoalveolar lavage fluid from non-small cell lung cancer patients [77]. Filter clogging is a major problem to be solved in ultrafiltration. Davies et al. developed a microfluidic filtration system by either pressure-driven or electrophoresis-driven isolation of EVs. Pore size of the filter could be controlled during device fabrication. EVs were separated from whole blood using the microfluidic device. DC electrophoresis was employed to propel EVs across the filter, while sample and collection streams were injected at equal flow rates in respective channels. Thus, clogging of pores with larger particles and cells from the bulk stream was prevented [78].

**Figure 4:**
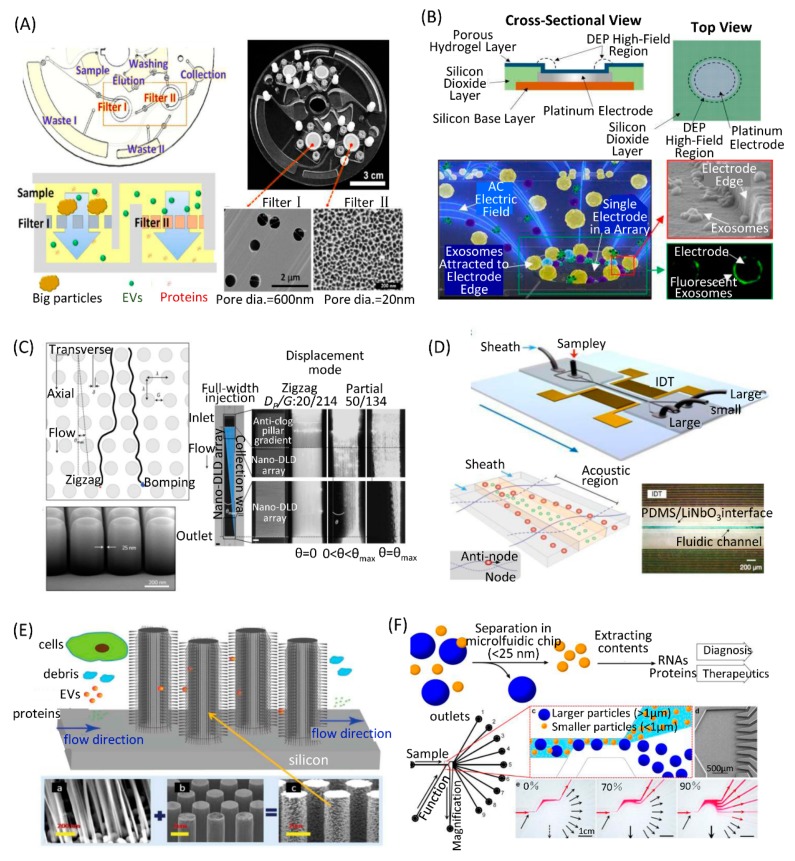
Size-dependent separation EV on microfluidic chips. (**A**) A lab-on-a-disc integrated with two nanofilters to separate EVs. Reprinted with permission [80]. Copyright 2017, ACS Publications. (**B**) EV separation by the dielectrophoretic separation forces generated by ACE microarray on a chip. Reprinted with permission [81]. Copyright 2017, ACS Publications. (**C**) EV separation by DLD pillar arrays on a chip. Reprinted with permission [84]. Copyright 2016, Nature Publishing Group. (**D**) EV separation by ultrasound waves on a chip. Reprinted with permission [89]. Copyright 2017, NAS Publishing Group. (**E**) EV separation by ciliated micropillars on a chip. Reprinted with permission [90]. Copyright 2013, RSC Publishing Group. (**F**) EV separation by viscoelastic forces on a chip. Reprinted with permission [92]. Copyright 2017, Nature Publishing Group.

Double membranes with small and large pore sizes could be assembled in a microfluidic device to separate EVs. Liang et al. developed an integrated double-filtration microfluidic device based on size-exclusion to enrich small EVs. Two membranes with pore sizes of 200 nm and 30 nm in diameter were embedded in the device. EVs larger than 200 nm were excluded by the 200 nm-pore-size membrane and EVs smaller than 30 nm passed through the device to the waste chamber. EVs with a size between 30–200 nm were separated and concentrated in the isolation chamber. EV separation and detection were integrated in the microfluidic device. Enriched EVs in the isolation chamber were labeled with biotinylated anti-CD63 antibodies and detected by enzyme-linked immunosorbent assays (ELISA) [79]. Woo et al. presented a lab-on-a-disc integrated with two nanofilters to separate EVs (Figure 4A). Raw biological samples without pre-treatment were used. Automated concentration of EVs in the size range of 20 nm to 600 nm was achieved within 30 min using a tabletop-sized centrifugal microfluidic system. It was confirmed that the device enabled >95% recovery of EVs from cell culture medium. In addition, the device provided >100-fold higher concentration of mRNA as compared with the ultracentrifugation method [80].

#### 3.2.2. Dielectrophoretic Isolation

Dielectrophoresis is known to provide effective separations of cells and cellular nanoparticles from clinical and biological samples. Ibsen et al. developed an alternating current electrokinetic (ACE) microarray chip to rapidly separate EVs from undiluted human plasma samples (Figure 4B). An alternating current electric field was applied to create the dielectrophoretic separation force in the ACE microarray chip. Nanoparticles are attracted to the dielectrophoretic high-field regions around the circular microelectrode edges. Cells and larger particles are pulled into the dielectrophoretic low-field regions. A small sample volume (30–50 μL) was required. EVs were concentrated into the high-field regions within 15 min, while other components in plasma were washed away with buffer. Subsequent on-chip immunofluorescence detection of EV proteins could be performed. The entire isolation process and on-chip fluorescence analysis was completed in less than 30 min [81]. Lewis et al. applied this method for capture and analysis of EVs directly from whole blood, plasma, or serum; pretreatment or dilution of the sample is not required. Subsequent on-chip immunofluorescence analysis permits specific identification and quantification of target biomarkers within as little as 30 min total time [82]. Chen et al. developed a simple dielectrophoretic method for EV isolation. The device contained an electrode layer, a PDMS microfluidic layer, the fixture, the lid, and the polymethyl methacrylate (PMMA) bottom. To reduce the degradation by electrochemical reactions, electrodes were covered with poly-HEMA to avoid direct contact between EVs and the high conductivity plasma sample. The edges of the microelectrodes showed high electric field intensity. They demonstrated higher recovery efficiency (>83%) and higher purity of EVs separated by the dielectrophoretic method than ultracentrifugation. Using the microfluidic chip, they separated EVs from the plasma of both lung cancer patients and healthy controls. They found that EVs from lung cancer patient plasma samples contained higher levels of miR-21, miR-191, and miR-192 compared to those from healthy controls. With on-chip detection, EGFR in EVs could distinguish lung cancer patients from healthy controls [83]. 

#### 3.2.3. Deterministic Lateral Displacement

Deterministic lateral displacement (DLD) pillar arrays are an efficient technology to separate micrometer scale particles. DLD has emerged as a potential lab-on-a-chip platform for biocolloid separation and on-chip diagnostics [94,95]. DLD can be fabricated using a silicon-based approach and applied to biocolloids at the nanoscale. Benjamin et al. used silicon processes to produce nanoscale DLD (nano-DLD) arrays of uniform gap sizes ranging from 25 to 235 nm (Figure 4C). A syringe pump was used to drive samples into the flow through nano-DLD arrays. They demonstrated that the nano-DLD arrays separated nanoparticles between 20 to 110 nm based on size with sharp resolution. Furthermore, they demonstrated size-based displacement of EVs in the nano-DLD, suggesting that biological nanoparticles can be operated in the platform [84]. After that, they integrated 1024 nano-DLD arrays on a single chip. It was capable of parallel processing sample fluids at rates of up to 900 μL per hour. They compared the nano-DLD chip with commonly used EV isolation technologies, including ultracentrifugation, ultracentrifugation plus density gradient, size-exclusion chromatography, and the exoEasy Maxi Kit. They claimed a superior yield for both serum and urine samples by their method. Further, RNA sequencing was carried out on nano-DLD- and ultracentrifugation-isolated EVs from prostate cancer patient serum samples. They found a higher gene expression correlation between replicates for nano-DLD-isolated EVs with enriched miRNA, decreased rRNA, and the ability to detect previously reported RNA indicators of aggressive prostate cancer. Thus, nano-DLD might be a promising alternative technology for fast and reproducible EV separation [85].

Zeming et al. developed a large-pore (2 μm) DLD device to separate nanoparticles (51–1500 nm) in real-time. They investigated innate long-range electrostatic influences on nanoparticles within a fluid medium at different NaCl ionic concentrations. They demonstrated that electrostatic forces cannot be assumed as negligible, especially for precise nanoparticle separation methods, such as DLD. They established a model to simultaneously quantify and modulate the electrostatic force interactions between nanoparticles and micropores. They achieved dynamic nanoparticle size separation on a single device with a rapid response time (<20 s) and an enlarged dynamic range (>1200%) by controlling buffer solutions. The system was driven by a negative pressure at the outlet. Sample and buffer inlets are open-air reservoirs that facilitate rapid changes of the ionic buffer inputs. This device provided opportunities for high-throughput applications in nano-separation for industrial and biological applications [86]. 

#### 3.2.4. Acoustic Purification

Acoustics-based microfluidics uses ultrasound waves to exert size-dependent forces on cells or particles for on-chip separation. A major difficulty in acoustic separation of EVs is the requirement for high radiation force, arising from the small size and low compressibility of EVs. Lee et al. presented an acoustic nano-filter system that separates EVs size-dependently in a continuous and contact-free manner. The separation is based on ultrasound standing waves that exert differential acoustic force on EVs according to their size and density. They achieved a high separation yield and resolution of large EVs (200–1000 nm) by optimizing the ultrasound transducer design and underlying electronics. The size cutoff could be controlled electronically in situ and enabled versatile EV-size selection. They successfully separated small EVs (<200 nm) from cell culture media, as well as large EVs in stored red blood cell products, using this device [87].

Evander et al. presented a microfluidic device based on microscale acoustic standing wave technology for rapid, non-contact capture of EVs in small sample volumes. The piezoelectric transducer was mounted on a printed circuit board to enable actuation through a waveform generator. The 2 × 0.2 mm^2^ capillary was clamped to the surface of the transducer and coupled with a thin layer of glycerol. The thin glass walls and small transducer resulted in a localized standing wave that trapped and held cells and nanoparticles in the center of the capillary above the transducer. Capture of platelet-derived microparticles from plasma was confirmed by scanning electron microscopy and flow cytometry. They analyzed the levels of plasma platelet-derived microparticles in 6 patients with ST-elevation myocardial infarction (STEMI) and 6 healthy controls using acoustic trapping method and compared the results with the standard differential centrifugation method. A significant increase in platelet-derived microparticles in patients compared to healthy controls was measured using both methods. The acoustic system offered a quick and automated setup for extracting EVs from small sample volumes with high recovery [88]. 

Wu et al. reported an acoustic-based microfluidic chip to separate EVs directly from whole blood in a label-free and contact-free manner (Figure 4D). This platform consisted of two modules: a microscale cell-removal module that first removed larger blood components, followed by EV subgroup separation in the EV separation module. They demonstrated the separation of 110-nm particles from a mixture of micro- and nanosized particles with a yield greater than 99% in the cell-removal module. They separated small EVs from an EV mixture with a purity of 98.4% in the EV separation module. Integrating the two modules onto a single microfluidic chip, they separated small EVs from whole blood with a blood cell removal rate of over 99.999%. The presence of EVs in the separated EV was confirmed by high expression of biomarkers, including HSP90, HSC70, CD63, and TSG101. The results further confirmed mRNA and miRNA expression in separated EVs [89].

#### 3.2.5. Others

In addition to the ones introduced above, researchers have developed other label-free microfluidic approaches to separate EVs based on their sizes without any recognizable damage. Wang et al. presented a microfluidic device consisting of ciliated micropillars, forming a porous silicon nanowire-on-micropillar structure (Figure 4E). This device can preferentially trap exosome-like lipid vesicles, while simultaneously filtering out proteins and cell debris. Trapped lipid vesicles can be recovered intact by dissolving the porous nanowires in PBS buffer [90]. Liu et al. developed a viscoelasticity-based microfluidic system to directly separate EVs from cell culture media or serum. A high separation purity (>90%) and recovery (>80%) of EVs was achieved by adding a small amount of biocompatible polymer into the media to control the viscoelastic forces exerted on EVs. The separation efficiencies of these 100 nm and 500 nm particles were demonstrated to be above 90% [91]. Shin et al. developed a microfluidic device that was composed of two inlets (Sample and Function channel), nine outlets, and a magnification channel (Figure 4F). The Sample and Function flows came in contact inside the channel. Nanoparticles were aligned through the upper wall. Larger particles moved toward outlets near the magnification channel, while smaller ones moved to upper outlets when the channel width was broadened. The magnification channel drew part of the Function flow, while the remaining Function and Sample flows flew to their respective outlets. By controlling the ratio of withdrawn flow to overall flow through the magnification channel, small EVs were separated through channel #1 to #3 and larger EVs were collected in outlets #5 to #9 [92].

The zeta potential of the EVs is reported to be negative around −12 mV. This allows exosomes to be separated by an electrophoresis. Marczak et al. reported a technique that utilized microfluidic gel electrophoresis and an ion-selective membrane to separate and concentrate EVs from a continuously flowing sample. The use of an electric field to separate EVs was reasonable as the electrophoresis of EVs is independent of its size, as their dimension is much larger than the Debye length. The authors operated the device at high transverse electric field strength (~100 V/cm) and various flow rates. EVs in samples were driven into the gel by electric forces and concentrated at the ion-selective membrane. They efficiently isolated EVs from PBS buffer, cell culture media, and blood. Nanoparticle tracking measurement revealed that the sizes of separated EVs were in the range of 60 to 130 nm [93].

### 3.3. EV Analysis on Microfluidic Chips

Microfluidic techniques allow the integration of EV separation and analysis on a single chip. In this section, we only discuss on-chip EV analysis. To confirm the size and morphology of separated EVs directly on chips, scanning electron microscopy and atomic force microscopy are generally performed [60,61,62,64,68,70,71,72,73,78,80,85]. However, both methods are not suitable for clinical application due to the complicated sample preparation steps and expensive machines. Fluorescent imaging is the most common way to detect and quantify EVs on microfluidic chips. The captured EVs can be stained with fluorescence dyes that specifically stain EV membranes [60,73], or with fluorecein-labeled antibodies [61,65,66,67,71,72,73,81,82,83]. On-chip ELISA is another common method to quantitatively detect the expression of EV biomarkers [62,69,76,80]. 

Some special microfluidic-based techniques have been developed to analyze EVs on chips. Im et al. presented a nPLEX sensor based on optical transmission through periodic nanoholes. The probing depth of these nanoholes was less than 200 nm, which is readily matched to small EV size for improved detection sensitivity. EVs were captured by antibodies conjugated to the sensor surface. The nPLEX sensor operated in a transmission mode that was able to detect the change of refractive index when EVs were captured. EVs captured on the nPLEX sensor were further targeted with anti-CD63 Au nanospheres or star-shaped particles to enhance spectral shifts [60]. Xu et al. integrated isolation and electrochemical analysis of EVs on a single chip. They designed a label-free and immobilization-free electrochemical aptasensor (named LGCD) which contains a CD63 aptamer and G-rich mimicking DNAzyme sequence. The single-stranded DNA forms a hairpin structure and a DNAzyme mimicking sequence was caged in the stem-loop structure. The original single stranded DNA hairpin could be opened by CD63-positive EVs to form a G-quadruplex as a signal reporter by hemin. The hemin/G-quadruplex could be employed as the Reduced form of nicotinamide adenine dinucleotide (NADH) oxidase and horseradish peroxidase (HRP)-mimicking DNAzyme simultaneously. Hence, the newly generated H_2_O_2_ by NADH oxidation could be catalyzed continuously and was coupled with great signal enhancement. The electrochemical sensor could detect CD63 positive-EVs at an extremely low concentration (4.39 × 10^3^ particles/mL) [74]. 

To analyze EV-associated RNA on chips, Shao et al. presented a microfluidic platform with three functional compartments: an EV enrichment chamber, RNA extraction chamber, and RNA analysis chamber. They used immunomagnetic beads coated by anti-EGFR or anti-EGFRvIII antibody to capture EVs from GBM patients’ blood samples. Captured EVs were lysed and mRNA contents were analyzed by on-chip reverse transcription polymerase chain reaction (RT-PCR) in the RNA analysis chamber [70]. Taller et al. developed a new on-chip analysis strategy for the rapid lysis of EVs and the detection of the released miRNA. A lysis chip and a detection chip were developed. EV-associated RNA was released based on surface acoustic wave, then RNAs were detected by an ion-exchange nanomembrane that consisted of an anion-exchange nanoporous membrane sandwiched between sensing and counter reservoirs. RNA in the sensing reservoir hybridized to complimentary oligos immobilized on the membrane surface. Current–voltage characteristics dramatically changed when negatively charged large molecules, such as RNA, were adsorbed to the surface of the positively charged membrane. The detection limit of the ion-exchange nanomembrane sensor was 2 pM [96].

## 4. Microfluidic Chip-based Liquid Biopsy

EVs in biofluid can be a source of quantitative and qualitative information [3,5]. Quantitative information comprising EV numbers can indicate the presence of malignant disease and tumor burden. Qualitative information through the molecular characterization of EV contents, including proteins and nucleic acids, can assist molecular classification and prognostic assessment of cancers and even guide precision therapy. In this section, we will discuss the application of microfluidics in EV-based liquid biopsy of cancer (Table 2).

### 4.1. Breast Cancer

Vaidyanathan et al. applied a tunable alternating current electrohydrodynamic microfluidic device to separate EVs from cell culture medium and patient serum. The device was functionalized with anti-HER2 or anti-CD9 capture antibody. Captured EVs were detected by a simple and rapid on-chip naked eye detection readout based on the catalytic oxidation of peroxidase substrate 3,3′,5,5′-tetramethylbenzidine (TMB). A higher level of HER2(+) EVs was detected in the HER2(+) patient serum compared to the HER2(−) patient serum using this device [69]. Kang et al. developed a dual-patterned immunofiltration microfluidic chip for EV capture. It is notable that the captured EVs could be released by dissociation of the cleavable linkers between capture antibody anti-CD63 and solid surface. The captured EVs were detected with fluorescent dye conjugated to anti-EpCAM antibody on the chip. They used this platform to discriminate the EpCAM(+) EVs level in serum between 2 breast cancer patients and 5 healthy individuals. They found that EVs from breast cancer patients expressed significantly higher level of EpCAM [68]. Fang et al. presented a microfluidic device in which EVs could be captured by anti-CD63 immunomagnetic beads. The captured EVs were immunolabeled and quantified with tumor associated antibodies (anti-EpCAM and anti-HER2). They demonstrated a significantly higher level of EpCAM(+) EVs in breast cancer patient plasma than in healthy controls. Importantly, they found that the levels of HER2+ EVs were consistent with HER2 expression in tumor tissues assessed by immunohistochemical staining. This suggests that their platform may provide an alternative method for molecular classification of breast cancer patients [73]. Liu et al. developed a droplet microfluidic chip for digital qualification of target EVs. They applied this platform to achieve quantitative detection of EVs in plasma samples from breast cancer patients. They found that the average Glypican-1(+) EVs in the breast cancer group (12 patients) increased by five- to seven-fold compared to the control groups (5 healthy sample and 5 patients with benign breast disease). In addition, the levels of Glypican-1(+) EVs were significantly decreased in two patients after surgery [76].

### 4.2. Ovarian Cancer

Im et al. developed a microfluidic chip for label-free and high-throughput EV analyses based on SPR. This device was functionalized with CD63 or CD24 to capture EVs from ovarian cancer patient ascites (n=20) and noncancer patients (n=10). They analyzed cancer-associated biomarkers (EpCAM, CD24, CA-125, CA19-9, HER2, mucin 18; EGFR, claudin 3) and noncancer-associated biomarkers (CD45, CD41, and D2-40). They found that EV-associated EpCAM and CD24 were significantly higher in the ovarian cancer patients than in controls [60]. Zhao et al. separated EVs from plasma using immunomagnetic beads coated with different antibodies on a microfluidic chip. They demonstrated a 3–5-fold increase in EV-associated expressions of EpCAM, CA-125, CD9, CD81, and CD63 in ovarian cancer patients compared to healthy controls. In addition, significantly increased expression levels of EV-associated CD24, EpCAM, and CA-125 were found in ovarian cancer patients compared to healthy patients. The chip-based plasma EV assay highly discriminated ovarian cancer patients from healthy individuals [72]. Zhang et al. utilized a microfluidic platform with a graphene oxide/polydopamine (GO/PDA) interface for EVs capture and analysis. This platform was able to efficiently capture EVs by anti-CD81 antibody and discriminate plasma EVs derived from ovarian cancer patients (n = 7) from the healthy controls (n = 5) by the mixture containing three biomarkers (CD63, CD81, and EpCAM). In addition, they found a ~10-fold decrease in the expression levels of the three markers for the post-treatment samples compared to the levels at the time of diagnosis [62]. Hisey et al. applied a herringbone-grooved microfluidic device coated with anti-CD9 or anti-EpCAM antibodies to capture EVs from high-grade serous ovarian cancer serum. They found that the amount of both total and EpCAM(+) EVs increased with disease progression from healthy, benign, stage I, to stage IV patients. High-grade serous ovarian cancer patients had a significantly larger number of EpCAM(+) EVs than the benign controls in serum [66].

### 4.3. Lung Cancer

Liu et al. utilized a microfluidic ultrafiltration chip to isolate small EVs from non-small cell lung cancer (NSCLC) patients. Various clinical samples, including plasma, urine, and lung bronchoalveolar lavage fluid, were tested on the chip. They found that EVs from plasma were smaller and more abundant than EVs from bronchoalveolar lavage fluid and urine. Quantification of total RNA isolated from EVs demonstrated that RNA quantities were lowest in plasma EVs and highest in urine EVs, although plasma yielded the highest EV quantity. However, EVs separated from plasma might be contaminated with high-density lipoproteins, which have similar sizes to those of small EVs and are abundant in plasma. By contrast, the level of high-density lipoproteins is normally very low in urine. Size-dependent EV isolation cannot exclude high-density lipoproteins from plasma samples [77]. He et al. presented a microfluidic device and immunomagnetic beads to isolate and detect the EVs from plasma of NSCLC patients. Immunomagnetic beads coated by different antibodies (anti-EpCAM; anti-α-Insulin Growth Factor Receptor (IGF-1R); anti-CA125; anti-CD9; anti-CD81 and anti-CD63) were used to capture EVs. They found that the amount of EVs derived from plasma of cancer patients significantly increased compared to the healthy controls. Quantitative analysis demonstrated significant overexpression of IGF-1R in EpCAM(+) EVs from NSCLC patients compared to 6 healthy controls [71]. Dong et al. developed a herringbone and bionic microvilli structure microfluidic device to immunocapture tEVs from the blood of NSCLC patients. The chip contained the anti-EpCAM coated silicon nanowire arrays to mimic the structures of intestinal microvilli to increase the contact area and enhance the efficiency of EV capture. Reverse transcription Droplet Digital PCR (ddPCR) assay was used to quantitatively analyze RNA expression and monitor dynamic changes of ROS1 rearrangements and EGFR T790M mutations. Using the NanoVilli Chip-enriched EVs, they found 18 to 468 copies of the CD74-ROS1 rearrangement in 7 patients at diagnosis and 0 to 225 copies of acquired EGFR T790M mutation in 6 patients at their time of disease relapse. In the control studies, all of the 9 healthy donors were negative for both ROS1 rearrangement and EGFR T790M mutation [67]. Lim et al. developed a novel immuno-magnetic microfluidic chip with elongated magnetic nanowires for efficient isolation and detection of EVs. The nanowires were doped with magnetic nanoparticles and biotin moieties conjugated with diverse EV-specific antibodies (anti-CD9, anti-CD63, and anti-CD81). The captured EVs were stained by fluorescent carbocyanine dye DiO. They found that EV protein levels from cancer patients (7 breast cancer and 5 lung cancer) showed a 3.9-fold increase compared to the healthy controls (3 healthy individuals). In addition, EV-associated miR-21 from the plasma of lung cancer patients demonstrated the distinctly high expression level [97].

### 4.4. Pancreatic Cancer

Lewis et al. used an ACE microarray chip to separate EVs from plasma, serum, or whole blood of pancreatic ductal adenocarcinoma patients. By detection of glypican-1 and CD63, 20 patient samples could be distinguished from 11 healthy controls with 99% sensitivity and 82% specificity. The speed and simplicity of EV capture and biomarker detection on the ACE chip using whole blood enables “sample-to-answer” liquid biopsy [82].

Taller et al. developed a new on-chip analysis strategy for the rapid lysis of EVs and the detection of the released miRNA. Cell culture medium from pancreatic cancer cell lines was tested on the chips to detect hsa-miR-550 [96]. Ko et al. developed a microfluidic chip with antibody-coated magnetic nanopores (anti-CD9, anti-CD63, anti-CD81, and anti-EpCAM) to detect and analyze blood samples. In addition, linear discriminant analysis (LDA) was applied to detect different EV genes (CD45, CK18, CK19, Erbb3, glyceraldehyde phosphate dehydrogenase (GAPDH), Lgals1, AsPc1, BxPC3, HPAFII, MiaPaCa2, Panc1) to discriminate between different disease states (precancerous lesions, pancreatic cancer, and the healthy controls) based on a machine learning algorithm. The EV-associated mRNA signature is associated with specific disease states. Several genes (e.g., CD63) were differentially expressed between groups, but no single gene was able to classify individual patients into the correct groups due to the variance in expression among patients within groups [75].

Kanwar et al. developed a simple microfluidic chip functionalized with anti-CD63 antibody to capture EVs. They quantitatively analyzed EVs with a membrane fluorescent dye (DiO) by using a standard plate-reader. Ten independent experiments using detecting serum (5 pancreatic cancer patients and 5 healthy individuals) showed an overall increase of 2.34 (± 0.31) times the amount of EVs in cancer patients compared to healthy individuals. EVs derived from pancreatic cancer patients had increased levels of both CD63 (3.17 fold) and Rab5 (1.75 fold) proteins. In addition, the chip enabled recovery of EVs with intact RNA for further analysis. They found that 90 miRNAs were differentially expressed in EVs of pancreatic cancer patients in comparison to healthy control [59].

### 4.5. Glioblastoma Multiforme (GBM)

Chen et al. first presented an approach to immunocapture EVs from blood samples based on a microfluidic chip. EVs could be captured in microchannels coated with anti-CD63 antibodies, and RNAs could be extracted from these captured EVs. They found more EVs and RNA contents in the GBM patient serum compared to the normal serum. A point mutation in the IDH-1 mRNA was recognized in a subset of GBM patients [64]. Shao et al. developed a microfluidic platform-integrated EV enrichment chamber, RNA extraction chamber, RNA reservoir, and qPCR chamber. They used immunomagnetic beads coated with anti-EGFR antibody to capture EVs from GBM cell culture medium (SKMG3 and GLI36vIII). They selected three categories of mRNA targets: diagnostic markers, namely EGFR, podoplanin (PDPN), and ephrin type-A receptor 2 (EPHA2); drug resistance markers, namely O6-methylguanine DNA methyltransferase (MGMT), alkylpurine-DNA-N-glycosylase (APNG), glutathione S-transferase (GSΠ1), excision repair cross-complementation (ERCC1 and ERCC2), major vault protein (MVP), ATP-binding cassette/multidrug resistance-associated protein (ABCC3), caspase-8 (CASP8), and insulin-like growth factor binding protein 2 (IGFBP2); and a generic EV marker, namely CD63. The RNA contents in EVs were extracted and adsorbed by glass beads. Furthermore, they chose MGMT and APNG to analyze EV mRNA levels. The results showed the levels of EPHA2 and EGFR were significantly higher in GBM patients than in healthy donors, while PDPN levels were similar in both groups. They also found that the EV-associated MGMT mRNA levels were significantly higher in GBM patients and MGMT mRNA levels in EVs correlated with MGMT DNA promoter methylation. The mRNA levels of diagnostic markers (EPHA2, EGFR, and PDPN) in EVs were heterogeneous among clinical samples (n = 17). The accuracy increased to 90% when the diagnosis was based on combined markers [70].

Reátegui et al. developed a high-throughput chip coated with a cocktail of antibodies to capture EVs in GBM patient serum (2 patients) or plasma (11 patients) and 6 healthy controls. The total number of EGFRvIII copies was quantified by ddPCR. Using the GBM patient samples, the chip demonstrated high specificity for the EGFRvIII mutation in their patients. They demonstrated that EGFRvIII mutation was one key driver of genetic mutation of the GBM, but not all cells—even in positive tumors—showed EGFRvIII mutation. Thirty-eight cancer-associated genes, not previously reported in GBM patient EVs, were identified [65].

### 4.6. Others

Liang et al. developed an integrated double-filtration microfluidic device to enrich small EVs. Using the device, they found that the concentration of urinary EVs was significantly elevated in bladder cancer patients (n = 16) compared to healthy controls (n = 8). This integrated EV double filtration device had a sensitivity of 81.3% at a specificity of 90% in diagnosis of bladder cancer [79]. Woo et al. utilized a lab-on-a-disc integrated with two nanofilters to separate EVs from bladder-cancer patients. On-disc enzyme-linked immunosorbent assay showed high levels of CD9 and CD81 expression. This suggested that this method may be potentially useful in clinical settings to test urinary EV-based biomarkers for cancer diagnostics [80]. Ji et al. presented a single-cell EV capture and detection microfluidic platform based on immunoaffinity approach. Based on this high-throughput multiplexed platform, they revealed that the decrement of certain EV phenotypes (e.g., CD63+EV) was associated with the invasive feature of both oral squamous cell carcinoma (OSCC) cell lines and primary OSCC cells [61]. Lewis et al. used an ACE microarray chip to separate EVs from plasma of colon cancer patients. Elevated Glypican-1 was observed for metastatic but not for nonmetastatic disease [82].

## 5. Conclusions and Future Perspectives

The potential of EVs in cancer diagnosis, molecular classification, and monitoring treatment response have aroused a great deal of interest in the medical and bioengineering fields. Microfluidic-based EV separation and analysis reduce sample volume and time and enable high throughput EV analysis on a single chip. A large number of samples or a variety of parameters of a single sample can be analyzed simultaneously. The microfluidic technique provides a rapid and cheap “sample-to-result” solution to facilitate the development of point-of-care instruments in liquid biopsy of cancer.

However, both surface biomarker- and size-dependent microfluidic approaches in EV separation have limitations. Although immunoaffinity separation based on biomarkers achieves a high purity and specificity, EV subtypes with low or negative expression of the proteins against capture antibodies will be lost during separation. Size-dependent separation may give rise to contamination with some nanoparticles with similar sizes to EVs in samples, such as high-density lipoprotein in plasma. The future microfluidic techniques need to realize high purity and yield of EV separation from various samples. EV lipid might be an ideal capture target, as it is universal in all EVs. In addition, automatic operation from sample loading to result reading is another challenge for the clinical application of microfluidic techniques.

Microfluidic-based EV manipulation is still an ongoing research subject. Besides the improvement of microfluidic platforms by bioengineers, standardized procedures of sample collection, storage, pre-treatment, and positive and negative criteria of tested biomarkers in diagnosis of various cancers need to be determined by both clinicians and biologists. Collaboration between scientists from different fields may accelerate microfluidic-based EV liquid biopsy in hospitals.

## Figures and Tables

**Table 1:** EV separation and detection on microfluidic chips. NA–not available.

Separation Strategy	Samples	Sample Pre-Treatment	Biomarkers	Particle Size (nm)	Detection Methods	Detection Limits	Ref.
Immunocapture via solid surfaces	Serum	No	CD63	30–300	Fluorescence imaging	0.5 pM	[59]
	Ascites	0.2 μm filter	CD63, CD24	~100	SPR	~3000 EVs (0.67 × 10^−3^ pM)	[60]
	Medium	No	CD63, CD9, CD81, EpCAM, HSP70	50–200	Fluorescence imaging	NA	[61]
	Plasma	10× dilution	CD63, CD81, EpCAM	70–230	ELISA	~50 EVs/µL	[62]
	Medium	No	CD63	<150	Fluorescence imaging	NA	[63]
	Medium, serum	0.8 μm filter	CD63, EV-associated total RNA	30–100	Scanning electron microscope (SEM), RT-PCR	NA	[64]
	Serum, plasma	Centrifugation and 0.8 μm filter	EGFR, EGFRvIII, Podoplanin, PDGFR	40–1000	Fluorescence imaging	100 EVs/µL	[65]
	Serum	10× dilution	CD9, EpCAM	<200	Fluorescence imaging	NA	[66]
	Plasma	No	EpCAM, ROS1, EGFR T790M	30–300	Fluorescence imaging, RT-ddPCR	NA	[67]
	Medium, plasma	Centrifugation	CD63, EpCAM	100–200	Fluorescence imaging	NA	[68]
	Serum	No	CD9, HER2	30–350	ELISA	2760 EVs/μL	[69]
Immunocapture via magnetic nanoparticles	Blood	0.8 μm filter	EV-associated mRNA	NA	RT-PCR	NA	[70]
	Plasma	No	EpCAM, CA125, IGF-1R	40–150	Chemifluorescence	IGF-1R 0.281 pg/mL; p-IGF-1R 0.383 pg/mL	[71]
	Plasma	No	CA125, EpCAM, CD24	<150	Fluorescence imaging	750 EVs/µL	[72]
	Medium, plasma	Centrifugation	CD63	<100	Fluorescence imaging	NA	[73]
	Medium, serum	Centrifugation	Phosphatidylserine	<150	Electrochemical sensor	4.39 EVs/µL	[74]
	Serum	No	CD63, CD9, CD81, EpCAM, EV-associated mRNA	149.7 ± 100	PCR, machine learning	NA	[75]
	Plasma	No	CD63, Glypican-1	30–150	Droplet digital ELISA	~10 EVs/µL	[76]
Nanoporous membrane	Medium, plasma, urine, bronchoalveolar lavage fluid	Centrifugation	EV-associated miRNA, EV proteins	30–200	NanoString, Liquid chromatography-mass spectrometry (LC-MS)	NA	[77]
	Blood	No	CD9, CD63, CD81	~150	RT-PCR	NA	[78]
	Urine	Centrifugation and 0.22 μm filter	CD63	30–200	ELISA	35.0 AU/mL	[79]
	Medium, urine	No	CD9, CD81, EV-associated mRNA	20–600	ELISA, RT-PCR	NA	[80]
Dielectrophoresis	Plasma, blood, serum	No	TSG101, CD63, Glypican-1	50–150	Fluorescence imaging	NA	[81,82]
	Plasma	4× dilution	Protein (CD81, EGFR); miRNA (miR-21, miR-191, miR-192); mRNA (GAPDH, CD81, EGFR)	~111	HPLC-MS, qRT-PCR	NA	[83]
DLD	Colloidal samples	No	NA	20–110	Fluorescence imaging	NA	[84,85]
	Particles	No	NA	50–1500	NA	NA	[86]
Acoustics	Medium, blood	No	NA	<200	Fluorescence imaging	NA	[87]
	Plasma	No	CD42a	300–1000	Flow cytometry	NA	[88]
	Blood	No	NA	110	NTA	NA	[89]
Ciliated micropillars	Mixture of BSA, liposomes and beads	No	NA	80–120	NA	NA	[90]
Viscoelastic forces	Medium, serum	No	NA	100–500	Fluorescence imaging	NA	[91]
Mechanical forces	Medium	Centrifugation	NA	30–100	Fluorescence imaging	NA	[92]
Electrophoresis	Medium, serum	Centrifugation	NA	60–130	Fluorescence imaging	NA	[93]

**Table 2:** Clinical applications of microfluidic technique on cancer liquid biopsy. NA–not available.

Cancer	Biofluid	Biomarkers	Clinical Values	Ref.
Breast cancer	Plasma	EpCAM	Diagnosis	[68]
	Serum	HER2, CD9	Molecular classification	[69]
	Plasma	EpCAM, HER2	Diagnosis, molecular classification	[73]
	Plasma, serum	Glypican-1	Diagnosis	[76]
Ovarian cancer	Ascites	CD24, EpCAM	Monitoring therapy	[60]
	Plasma	CA-125, EpCAM, CD24	Diagnosis	[72]
	Plasma	CD9, CD81, EpCAM	Diagnosis, monitoring therapy	[62]
	Serum	CD9, EpCAM	Diagnosis	[66]
	Plasma	EpCAM, IGF-1R, CA-125	Diagnosis	[71]
Lung cancer	Plasma, urine, bronchoalveolar lavage fluid	EV number	NA	[77]
	Plasma	EpCAM, IGF-1R	Diagnosis	[71]
	Plasma	ROS1 rearrangements, EGFR T790M mutation	Diagnosis	[67]
	Plasma	miRNA21	Diagnosis	[97]
Pancreatic cancer	Whole blood, plasma, serum	Glypican-1, CD63	Diagnosis	[82]
	Serum	CD63, Rab5, EV-associated miRNAs	Diagnosis	[59]
GBM	Serum	EV-associated total RNA	Diagnosis	[64]
	Serum	EV-associated mRNA (EPHA2, EGFR, PDPN)	Diagnosis	[70]
	Serum, plasma	EGFRvIII mutation	Diagnosis	[65]
Bladder cancer	Urine	CD63	Diagnosis	[79]
	Urine	CD9, CD81	Diagnosis	[80]
Colon cancer	Whole blood, plasma, serum	Glypican-1, CD63	Diagnosis	[82]
	Plasma	EpCAM	Diagnosis	[68]
